# *No-Cap* Flowable Bulk-Fill Composite: Physico-Mechanical Assessment

**DOI:** 10.3390/polym15081847

**Published:** 2023-04-11

**Authors:** Abdullah Alshehri, Feras Alhalabi, Ali Robaian, Mohammed A. S. Abuelqomsan, Abdulrahman Alshabib, Eman Ismail, Faisal Alzamil, Nawaf Alotaibi, Hamad Algamaiah

**Affiliations:** 1 Department of Conservative Dental Sciences, College of Dentistry, Prince Sattam Bin Abdulaziz University, Al-Kharj 11942, Saudi Arabia; 2 Department of Restorative Dental Science, College of Dentistry, King Saud University, Riyadh 11545, Saudi Arabia; 3 Engineer Abdullah Bugshan Research Chair for Dental and Oral Rehabilitation, King Saud University, Riyadh 11545, Saudi Arabia; 4 Department of Clinical Dental Sciences, College of Dentistry, Princess Nourah Bint Abdulrahman University, Riyadh 11564, Saudi Arabia; 5 College of Dentistry, Prince Sattam Bin Abdulaziz University, Al-Kharj 11942, Saudi Arabia

**Keywords:** bulk-fill resin-based composite, flexural strength, surface roughness, microhardness, color stability

## Abstract

(1) Background: A newer class of *flowable* bulk-fill resin-based composite (BF–RBC) materials requires no capping layer (Palfique Bulk flow, PaBF, Tokuyama Dental, Tokyo, Japan). The objective of this study was to assess the flexural strength, microhardness, surface roughness, and color stability of PaBF compared to two BF-RBCs with different consistencies. (2) Methods: PaBF, SDR Flow composite (SDRf: Charlotte, NC, USA) and One Bulk fill (OneBF: 3M, St. Paul, MN, USA) were evaluated for flexural strength with a universal testing machine, surface microhardness using a pyramidal Vickers indenter, and surface roughness using a high-resolution three-dimensional non-contact optical profiler, a and clinical spectrophotometer to measure the color stability of each BF–RBC material. (3) Results: OneBF presented statistically higher flexural strength and microhardness than PaBF or SDRf. Both PaBF and SDRf presented significantly less surface roughness compared with OneBF. Water storage significantly reduced the flexural strength and increased the surface roughness of all tested materials. Only SDRf showed significant color change after water storage. (4) Conclusions: The physico-mechanical properties of PaBF do not support its use without a capping layer in the stress bearing areas. PaBF showed less flexural strength compared with OneBF. Therefore, its use should be limited to a small restoration with minimal occlusal stresses.

## 1. Introduction

Resin-based composite (RBC) is the restorative material of choice for anterior and posterior direct restorations as indicated by the Academy of Operative Dentistry–European Section [1]. RBC materials are esthetic and durable restorative materials that can be used in several clinical conditions [2]. However, despite their improved physical and mechanical properties, RBC materials still have several shortcomings [3], such as inadequate depth of cure, the deterioration of mechanical properties, and color instability. In order to ensure the adequate degree of conversion (curing) of RBC materials, the incremental placement of RBC restorations is recommended [4,5]. However, this technique can be time-consuming, especially for large size restorations. In addition, it may result in contamination between RBC layers and the formation of voids within RBC restorations [4], which may impair the structural integrity of RBC restorations, resulting in the mechanical failure (fracture) of the RBC restorations. In addition, the inadequate depth of curing has prevented the use of conventional RBC thick layers (>2 mm) in various clinical cases [6], increasing the chair-side time required for the placement of large-size RBC restorations.

On the other hand, Bulk-fill RBCs (BF-RBCs) were introduced to the market with the goal of saving application time by using practical techniques [3,4]. BF-RBC materials allow for the placement of direct restorations in thick increments (up to 4 or 5 mm) and simplifies the restorative procedure [7]. Compared with conventional RBC materials, BF-RBCs have a higher degree of conversion, depth of cure, less polymerization shrinkage stress, and adequate marginal integrity. However, their mechanical and physical properties are material-dependent [4]. Despite the fact that some BF-RBCs may have adequate flexural strength [8], they exhibit lower hardness and elastic modulus compared to conventional RBCs [8]. BF-RBCs materials can be categorized according to their consistency as regular viscosity and low viscosity (flowable) materials [9]. Flowable BF-RBC materials usually require an additional capping later of high-viscosity RBC material, particularly in areas with high occlusal stress [8,9]. Such a capping layer is thought to reduce the wear rate and improve the mechanical performance of RBC restorations [9]. An additional reason for the replacement of RBC restorations is insufficient color stability [10], which impairs the esthetic outcome of RBC restorations. The staining of RBC materials can be caused by extrinsic or intrinsic factors [11]. External staining can be related to the adsorption or absorption of staining pigments from diet, plaque accumulation, and surface degradation [12]. Intrinsic factors, which cause the staining of the material in the absence of external influences, include the resin matrix ingredients, filler amount and size, and photo-initiator type [11,13]. Flowable RBC materials present more staining and less color stability compared with RBC of regular viscosity due to their low filler/resin ratio.

To simplify the placement of BF-RBCs even more, Palfique Bulk Flow (PaBF: Tokuyama Dental, Tokyo, Japan) has been marketed as a flowable BF-RBC material that can adapted to irregular cavities and requires no capping layer of regular-viscosity RBC. PaBF has numerous indications such as direct anterior and posterior restorations, cavity lining, the blocking out of cavity undercuts, and the repairing of the porcelain or RBC [14]. In addition, PaBF claims to allow a level of polymerization shrinkage stress, provides a high curing depth, excellent cavity adaptation, good surface properties, and causes less wearing of the opposing tooth due to superior characteristics of wear resistance [14,15]. BF-RBCs are based on different technologies, such as a stress decreasing monomer or intensified photo-initiators [16,17]. Radical Amplified Photopolymerization (RAP) catalyst technology and a proprietary filler technology are utilized in PaBF. Such technologies claim to enhance the degree of conversion (curing) and improve the surface properties of PaBF compared with BF-RBC materials utilizing different technologies [14]. The question is whether the physicomechanical properties of PaBF support its clinical use without a capping layer. Indeed, studies evaluating this recently introduced no-cap flowable BF-RBC are lacking. Therefore, the aim of this study is to conduct a thorough investigation to compare the physico-mechanical properties of PaBF to two BF-RBCs of different consistencies. The null hypotheses was that there would be no statistically significant differences between the (1) flexural strength, (2) microhardness, (3) surface roughness, and (4) color stability of the three BF-RBCs tested.

## 2. Materials and Methods

The materials used in the study, in addition to their compositions, are described in Table 1.

### 2.1. Flexural Strength Evaluation

**Figure 1:**
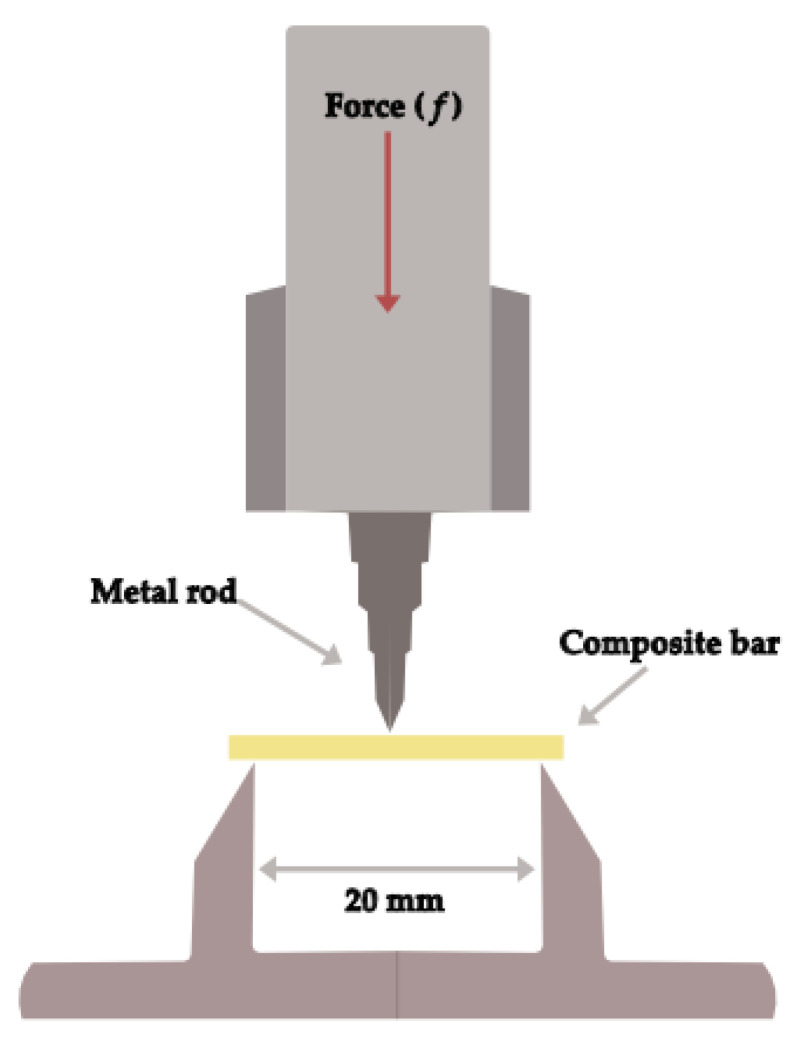
Schematic illustration of flexural strength evaluation.

A total of twelve specimens of each group were prepared using silicone molds (25 mm length × 2 mm width × 2 mm height) to prepare bar-shaped specimens (n = 6) at room temperature. Each RBC specimen was irradiated (light-cured) according to the manufacturer’s instructions in five overlapping points (40 s each) on the top surfaces starting from the periphery and moving sideways to the opposite end of the specimen using a (Elipar^TM^ S10, 3M ESPE, St. Paul, MN, USA) operated at 1000 mW/cm^2^ as verified by a hand-held radiometer (Bluephase Meter, Ivoclar Vivadent, Austria). The light-curing tip was kept at approximately 1 mm and at 0 angle to the specimen. Half of the specimens of each group were tested for flexural strength after storage in distilled water for 24 h at 37 °C, while the other half of the specimens were tested after storage in distilled water for 30 days at 37 °C. The flexural strength was evaluated using a three-point bending method with a 20 mm span and 1 mm/min cross-speed in a universal testing machine (an Instron 5965 testing machine; Norwood, MA, USA) (Figure 1). Prior to testing, the thickness of each specimen was measured using a digital micrometer (Mitutoyo, Tokyo, Japan) at the center and at each end of the test specimen. The load at fracture and specimen dimensions were used to calculate the flexural strength in MPa according to the following formula:

$$\sigma =\frac{3fl}{2bh^{2}}$$

*F* is the maximum force applied in Newtons, *l* is the distance between the two points of support as measured in millimeters, *b* is the width of the RBC specimen as measured in millimeters, and *h* is the height of the RBC specimen in millimeters.

### 2.2. Vicker’s Microhardness (VMH) Evaluation

**Figure 2:**
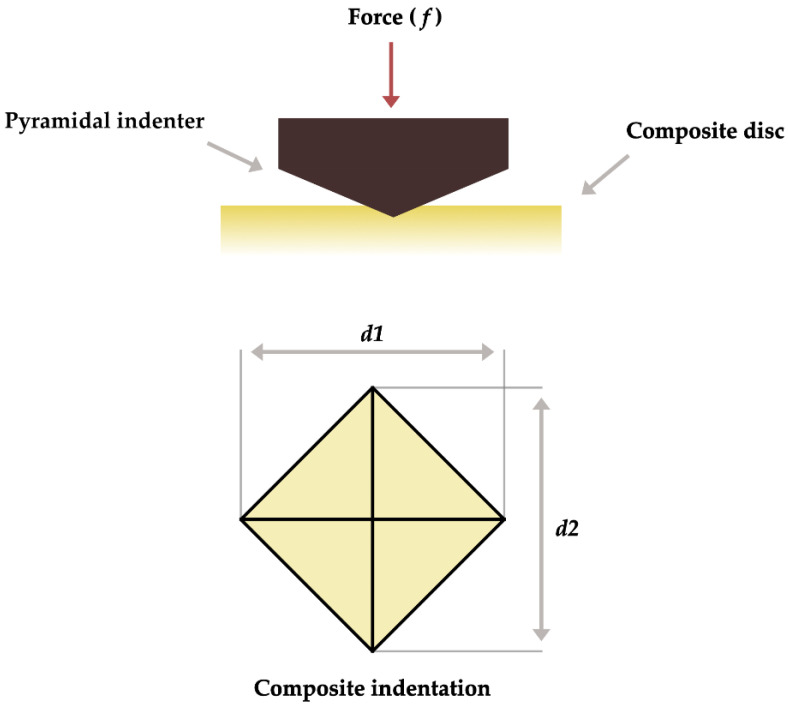
Schematic illustration of Vicker’s Microhardness (VMH) evaluation.

A total of 12 cylindrical (4 × 6 mm) BF-RBC specimens were prepared using a silicone mold on a polyethylene terephthalate strip and light cured according to the manufacturer’s instructions using a light-emitting-dione (LED) light curing unit (Elipar^TM^ S10, 3M ESPE, St. Paul, MN, USA) operated at 1000 mW/cm^2^ as verified by a hand-held radiometer (Bluephase Meter, Ivoclar Vivadent, Bürs, Austria). The light-curing tip was kept at approximately 1 mm and at 0 angle to the specimen. Each experimental group was stored in an incubator for 24 h at 37 °C and used for microhardness assessment. Half of the specimens of each experimental group (n = 6) were tested at 24 h, while the other half (n = 6) was tested after 30 days of water storage at 37 °C. Five indents were made using a pyramidal Vickers indenter (INNOVATEST Europe BV, Maastricht, The Netherlands) (Figure 2) with a 10-N load and a dwell time of 15 s. The indentations made were approximately 0.5 mm apart from each other. The size of each indentation was measured using a built-in microscope at ×50 and the Vickers hardness number (VHN) was calculated based on the following formula: VMH = 1.854(*f*/*d2*), while F is the force applied and *d2* is the surface area of the indentation. The five VMH measurements of each specimen were averaged to obtain a single mean.

### 2.3. Surface Roughness (Ra) Evaluation

BF-RBC material was inserted in a silicone mold of 2 mm thickness and 8 mm diameter on a polyethylene terephthalate strip, covered with another on a polyethylene terephthalate strip, and then light cured according to manufacturer’s instructions using a light-emitting-dione (LED) light curing unit (Elipar^TM^ S10, 3M ESPE, St. Paul, MN, USA) operated at 1000 mW/cm^2^ as verified by a hand-held radiometer (Bluephase Meter, Ivoclar Vivadent, Austria). The light-curing tip was kept at approximately 1 mm and at 0 angle to the specimen. BF-RBC samples (n = 6) were kept in distilled water for 24 h, then finished and polished using aluminum oxide discs (Sof-Lex, 3M, St. Paul, MN, USA) for 10 s for each disc. The kit has four discs (coarse, medium, fine, and superfine). Rinsing with water and air-drying were performed between each disc. Half of the specimens of each group were tested for surface roughness (Ra) after storage in distilled water for 24 h at 37 °C, while the other half of the specimens were tested after storage in distilled water for 30 days at 37 °C. The Ra of each RBC specimen was evaluated using a high-resolution three-dimensional (3D) non-contact optical profiler (Contour GT-K 3D Optical Microscope, Bruker^®^, Billerica, MA, USA). The specimens were vertically scanned at 5× Michelson magnification and at a field of view of 1 × 1 mm. The scan speed was 1×, and the thresholding was 4. The software used for the analysis and graphical output was Vision 64 (Bruker, Billerica, MA, USA). Four scans were obtained and averaged for each specimen.

### 2.4. Assessment of Color Change

A total of six samples of each group (material) were prepared using a silicone mold on a polyethylene terephthalate strip and light cured according to the manufacturer’s instructions using a light-emitting-diode (LED) light curing unit (Elipar^TM^ S10, 3M ESPE, St. Paul, MN, USA) operated at 1000 mW/cm^2^ as verified by a hand-held radiometer (Bluephase Meter, Ivoclar Vivadent, Austria). The light-curing tip was kept at approximately 1 mm and at 0 angle to the specimen. BF-RBC materials were packed into the mold and then covered by another polyethylene terephthalate strip and light-cured according to the manufacturer’s instructions. A clinical spectrophotometer (VITA Easyshade Advance 4.0, Vita Zahnfabrik, Bad Säckingen, Germany) was calibrated according to the manufacturer’s instructions and used to measure the color of each RBC specimen immediately following the light-curing according to the manufacturer’s instructions, and after the 30-day water aging. The spectrophotometer’s CIE-LAB values provide a numerical representation of a 3D measure of color as described in previous studies [18,19]. Readings of L*, a* and b* were performed three times against the same background (black) and the mean value used. Total color change was calculated using the following formula:

ΔE = (ΔL*2 + Δa*2 + Δb*2)1/2

### 2.5. Statistical Analysis

A Kolmogorov–Smirnov test was used to assess the data normality of the obtained results. A Kruskal–Wallis test and a Mann–Whitney test at a significance level of α = 0.05 were used to statistically analyze the flexural strength, surface roughness, and surface microhardness, and color change data were used to statistically analyze color stability data using the IBM Statistical Package for the Social Sciences (SPSS Inc., Chicago, IL, USA) version 22.

## 3. Results

### 3.1. Flexural Strength

**Figure 3:**
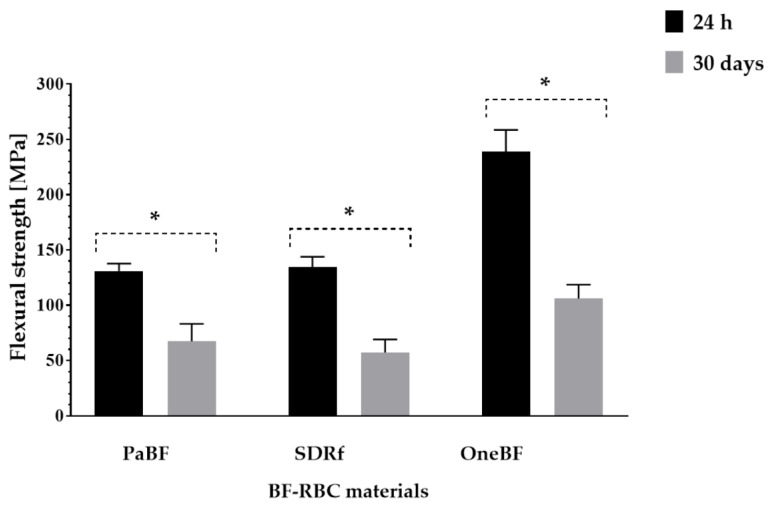
Comparison between the flexural strength of BF-RBC materials at 24 h and after 30 days of water storage. *: indicates statistically significant difference (*p* < 0.05) within each group.

The mean ± standard deviation (SD) and flexural strength (MPa) of BF-RBC materials is numerically described in Table 2. OneBF presented the highest flexural strength compared with PaBF at 24 h (*p* = 0.044) and after 30 days of water storage (*p* = 0.004), and compared with SDRf at 24 h (*p* = 0.002) and after 30 days of water storage (*p* = 0.024), and at 24 h and after 30 days of water storage. Water storage significantly reduced the flexural strength of the three BF-RBC materials (Figure 3).

### 3.2. Vicker’s Microhardness (VMH)

The mean ± SD VMH of BF-RBC materials is numerically described in Table 3. OneBF showed significantly higher VMH compared with SDRf at 24 h (*p* < 0.001) and after 30 days of water storage (*p* < 0.001). There was no statistically significant difference between VMH of PaBF and OneBF at 24 h and after 30 days of water storage. Water storage did not have a significant effect on the VMH of the three BF-RBC materials.

### 3.3. Surface Roughness (Ra)

**Figure 4:**
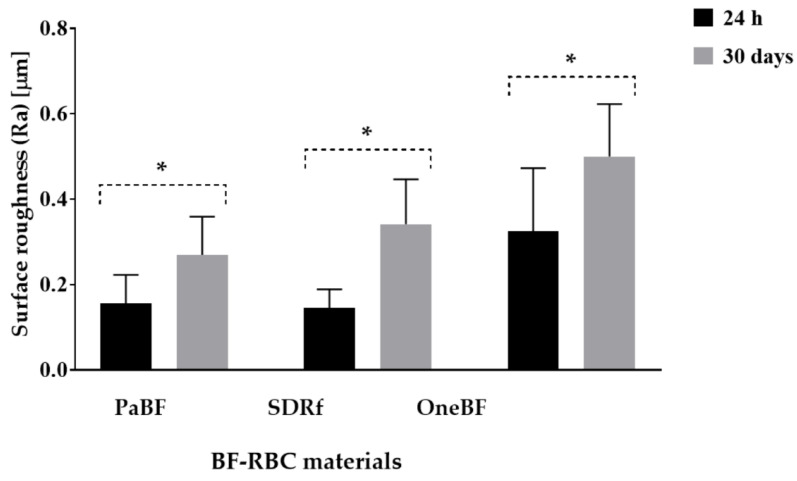
Comparison between the surface roughness (Ra) of BF-RBC materials at 24 h and after 30-days of water storage. * indicates a statistically significant difference (*p* < 0.05) within each group.

There was no statistically significant difference between the Ra of the three BF-RBC materials at 24 h (*p* = 0.050). However, after water storage, OneBF presented significantly higher Ra compared with PaBF (*p* = 0.012) (Table 4, Figure 4).

### 3.4. Color Change

OneBF showed significantly less color change compared with PaBF and SDRf (*p* < 0.001), while there was no statistically significant difference between the color change of the two flowable BF-RBC materials (SDRf and PaBF) (*p* = 0.136) or between SDRf and OneBF (*p* = 0.198) (Table 5).

## 4. Discussion

Thorough laboratory (in-vitro) evaluations of recently introduced dental restorative materials are required before clinical studies using such materials can be performed [20] in order to assess whether the physico-mechanical properties of no-cap bulk-fill flowable composite PaBF support its clinical use without a capping layer. This study was designed to evaluate the flexural strength, microhardness, surface roughness, and color stability of PaBF. PaBF was compared to two categories of bulk-fill composite materials; one that should be used clinically with a capping layer (SDRf), and the other can be used clinically without a capping layer (OneBF).

Dental restorations are usually subjected to a cyclic load during mastication (chewing). Mechanical failure (fracture) is one main cause of failure which decreases the clinical longevity of posterior RBC restorations [21]. This, in part, can be correlated with the mechanical properties of the RBC material used [22], such as flexural strength and microhardness. In this study, flexural strength, a bulk mechanical property of RBC, was evaluated, as it can estimate the resistance of RBC restorations to both compressive and tensile stresses simultaneously generated during chewing, which can then be used to characterize the mechanical properties of new restorative materials [23]. The forces of mastication cannot only lead to fracture, but also to the wearing of RBC restorations. Therefore, microhardness, a surface mechanical property of RBC, was assessed, as it can predict the wear resistance of BF-RBC restoration [24]. While microhardness can be assessed using different methods, the Vickers hardness method is recommended for RBC materials [25,26], and therefore it was used in this study.

It was hypothesized that there would be no statistically significant difference between the (1) flexural strength, and (2) microhardness of the three BF-RBCs tested. The results of this study indicate that the regular consistency of OneBF presented statistically higher flexural strength and microhardness than both the flowable composites (PaBF or SDRf) tested. Therefore, the first and second null hypotheses had to be rejected. It was assumed that increasing the filler content of the RBC materials would improve their mechanical properties and wear resistance [23]. The flexural strength of RBC materials is directly related to the filler content [27], which explains the significant difference between the mean flexural strength of OneBF with a filler content of 76.5 wt% and 58.4 vol% and SDRf with a filler content of 68 wt% and 45 vol%. Nevertheless, PaBF with a filler content of 70 wt% and 56 vol% presented less flexural strength compared to OneBF, but was higher than SDRf. Other factors such as the resin matrix composition might play a role in the RBC mechanical properties such as flexural strength [27,28]. The resin matrix of OneBF consists of aromatic urethane dimethacrylate (AUDMA), which is a high-molecular-weight monomer that minimizes the reactive groups in the resin chain; this enhances the rigidity of the final polymeric matrix upon the polymerization process [29,30]. Moreover, the addition-fragmentation chain transfer monomers (AFM) used in OneBF can improve polymer network homogeneity, which could lead to the enhanced mechanical properties of OneBF [31,32]. This finding is in accordance with recent studies in which OneBF presented superior flexural strength and microhardness [33,34] due to both its filler content and resin-matrix composition.

RBC surface properties such as surface roughness can affect the esthetic outcome of RBC restorations [35], which can be attributed to changes in color reflectance and stains accumulation onto RBC restorations. In addition, RBC restorations with a rough surface may be more associated with biofilm formation [36], which can result in marginal staining and further recurrent caries formation. It was hypothesized that there would be no statistically significant difference between the surface roughness of the three BF–RBCs tested. The results of this study indicate that both PaBF and SDRf presented less surface roughness compared with OneBF. Thus, the third hypothesis was rejected. The surface roughness of RBC materials may be more related to the composition of RBC material rather than other factors, such as polishing protocols [37]. Among the RBC materials tested, only PaBF contains monodispersing supra-sano spherical fillers [14], which would afford a smooth surface with less surface roughness. Meanwhile, the superior surface roughness of SDRf compared to OneBF can be explained by the reduced filler/resin ratio of SDRf. It has been reported that RBC with a low filler/resin ratio (low viscosity) may have less surface roughness [38]. It has been suggested that a surface roughness threshold of 0.2 µm is a critical value above which dental plaque accumulation might occur, favoring both caries and periodontal inflammation development [39]. 

RBC restorations are tooth-colored esthetic restorations. Intraorally, they are subjected to different fluids of variable pH and temperature. In fact, RBC properties such as surface roughness and color stability become altered over time [10]. Therefore, in order to simulate the intra-oral conditions, the tested BF-RBC materials were stored in water for 30 days to evaluate the internal color change. It is well known that water storage can result in the color changes of RBC materials [40]. It was hypothesized that there would be no statistically significant difference between the color stability of the three BF-RBCs tested. In this study, OneBF and PaBF presented adequate color stability compared with SDRf. Therefore, the fourth null hypothesis was also rejected. This finding can be explained by the filler/resin ratio of the three BF-RBC materials, as both OneBF and PaBF have more filler/resin ratio than SDRf. RBC materials with less filler/resin might show more color change over time [41], which may be due to RBC water absorption [23]. This study is limited by the lack of long-term artificial aging, which would have a more prominent effect on the mechanical properties of BF–RBC materials. Despite such limitations, the results of this study provide information on a recently introduced restorative material (*no-cap* flowable bulk-fill composite) used clinically. Further studies are recommended in order to evaluate the mechanical behavior of restorations made with OneBF and PaBF. 

## 5. Conclusions

The physico-mechanical properties of PaBF do not support the use of PaBF without a capping layer in occlusal stress-bearing areas. Therefore, PaBF use without a capping layer should be limited to a small restoration with minimal occlusal stresses. PaBF showed less flexural strength and microhardness, but showed less surface roughness. Further long-term evaluations of wear resistance and fracture toughness are required.

## Figures and Tables

**Table 1:** Materials used in the study.

Material (Manufacturer)	Code	Composition	Filler Load wt%/vol%.
Palfique Bulk flow (Tokuyama Dental, Tokyo, Japan)	PaBF	Bis-GMA, TEGDMA, Bis-MPEPP, Mequinol, Dibutyl hydroxyl toluene and UV absorber	70/56%
SDR Flow composite (Dentsply Sirona Charlotte, NC, USA)	SDRf	Modified UDMA, ethoxylated ethoxylated Bisphenol A dimethacrylate, and TEGDMA, resins. The filler is a combination of Bari-um-alumino-fluoro-borosilicate glass and strontium alumino-fluoro-silicate glass	68/45%
One Bulk fill (3M, St. Paul, MN, USA)	OneBF	Zirconia/ silica cluster, itterbium trifluoride, AUDMA, UDMA, DDDMAA, AFM	76.5/58.5%

Bis-GMA: Bisphenol-A glycidyl dimethacrylate, TEGDMA: Triethylene glycol dimethacrylate, Bis-MPEPP: Bisphenol A polyethoxy methacrylate, UDMA: Urethane dimethacrylate, AUDMA: aromatic urethane dimethacrylate, DDDMAA: 1, 12-Dodecanediol dimethacrylate, AFM: addition fragmentation monomer, Bis-EMA: Bisphenol-A ethoxylated dimethacrylate.

**Table 2:** Mean ± Standard deviation flexural strength of BF-RBC materials at 24 h and after 30 days of water storage.

Group	Flexural Strength (24 h) (Mean ± SD)	Flexural Strength (30 days) (Mean ± SD)	Flexural Strength Loss (%)
**PaBF**	130.97 ± 6.71 ^a^	67.51 ± 15.69 ^A^	48.45
**SDRf**	134.58 ± 9.26 ^a^	57.23 ± 11.96 ^A^	57.47
**OneBF**	238.82 ± 19.79 ^b^	106.17 ± 12.40 ^B^	55.54

Different superscript letters indicate a statistically significant difference (*p* < 0.05) between the groups at 24 h (small letters) and after 30 days of water storage (capital letters).

**Table 3:** Mean ± standard deviation Vicker’s microhardness (VMH) of BF-RBC materials at 24 h and 30 days of water storage.

Group	VMH (24 h) (Mean ± SD)	VMH (30 days) (Mean ± SD)	Microhardness Loss (%)
PaBF	36.2750 ± 0.59 ^a,b^	36.135 ± 0.37 ^A,B^	0.38
SDRf	18.45 ± 0.21 ^a^	18.41 ± 0.18 ^A^	0.21
OneBF	65.51 ± 1.40 ^b^	68.83 ± 2.35 ^B^	−5.06

Different superscript letters indicate a statistically significant difference (*p* < 0.05) between the groups at 24 h (small letters) and after 30 days of water storage (capital letters).

**Table 4:** Surface roughness (Ra) of BF-RBC materials at 24 h and after 30-days water storage.

Group	Surface Roughness (Ra) [μm] (24 h)	Surface Roughness (Ra) [μm] (30 days)	Surface Roughness Increase
**PaBF**	0.16 ± 0.07 ^a^	0.27 ± 0.09 ^A^	68.75
**SDRf**	0.15 ± 0.04 ^a^	0.34 ± 0.10 ^A,B^	126.6
**OneBF**	0.33 ± 0.15 ^a^	0.49 ± 0.124 ^B^	48.48

Different superscript letters indicate a statistically significant difference (*p* < 0.05) between the groups at 24 h (small letters) and after 30 days of water storage (capital letters).

**Table 5:** Mean ± Standard deviation color change of BF-RBC materials after 30 days of water storage.

Group	Color Change (ΔE)
**PaBF**	20.72 ± 2.565 ^A,B^
**SDRf**	11.55 ± 2.803 ^B^
**OneBF**	5.057 ± 1.664 ^C^

Different superscript letters indicate a statistically significant difference (*p* < 0.05) between the groups.

## Data Availability

The data presented in this study are available upon request from the corresponding author.
